# Pharmacological and Non-Pharmacological Advancements in Heart Failure Treatment

**DOI:** 10.31083/j.rcm2506230

**Published:** 2024-06-25

**Authors:** Chen Wang, Gaoshuang Fu, Xinnan Wang, Ning Li

**Affiliations:** ^1^Institute of Basic Theory for Chinese Medicine, China Academy of Chinese Medical Science, 100700 Beijing, China

**Keywords:** heart failure, heart failure with preserved ejection fraction, heart failure with reduced and mildly reduced ejection fraction, pharmacological and non-pharmacological treatment

## Abstract

Heart failure (HF) is a complex, life-threatening condition characterized by high mortality, morbidity, and poor quality of life. Despite studies of epidemiology, pathogenesis, and therapies, the rate of HF hospitalization is still increasing due to the growing and aging population and an increase in obesity in relatively younger individuals. It remains a predominant issue in the public health and the global economic burden. Current research has focused on how HF affects the entire range of left ventricular ejection fraction (LVEF), especially the three HF subgroups. This review provides a latest overview of pharmacological and non-pharmacological strategies of these three subgroups (HF with preserved ejection fraction, HF with reduced ejection fraction, and HF with mildly reduced ejection fraction). We summarize conventional therapies, investigate novel strategies, and explore the new technologies such as aortic thoracic stimulation and interatrial shunting devices.

## 1. Introduction

“Heart failure (HF) is a clinical syndrome with symptoms and/or signs caused by a structural and/or functional cardiac abnormalities and documented by elevated natriuretic peptide levels and/or objective evidence of pulmonary or systemic congestion [1]”. HF is a long-term condition that influences approximately 63 million people worldwide with an increasing prevalence in older age groups, reaching 4.3% among persons aged 65 to 70 years in 2012 and projected to increase steadily through year 2030 when the prevalence of HF could reach 8.5%. The mortality remains high and has been approximately 50% after 5 years. It is estimated that patients with HF have a shorter lifespan of about 1.1–2.3 years due to premature death or disability. Furthermore, hospitalizations due to HF account for 1–2% of all admissions, making hospitalization the main contributor to the costs associated with HF and a high rate of readmission approaching 25% [2]. Therefore, developing new, preventative, and reparative treatment strategies is necessary. HF is classified into three subtypes based on left ventricular ejection fraction (LVEF): heart failure with preserved ejection fraction (HFpEF, LVEF ≥50%), heart failure with reduced ejection fraction (HFrEF, LVEF ≤40%), and heart failure with mildly reduced ejection fraction (HFmrEF, LVEF 41–49%) [3, 4]. This review provides an overview of the recent pharmacological and non-pharmacological treatments for patients with the three HF subtypes.

We commonly used databases for HF research including PubMed/MEDLINE, Embase, ScienceDirect and Wiley and develop a well-defined search strategy using relevant keywords, which included “heart failure”, “heart failure with preserved EF”, “heart failure with reduced EF”, “novel therapies”, and “non-pharmacological treatments”. We combined these terms using appropriate Boolean operators (AND, OR) to create comprehensive search strings. We analyzed the findings of the studies and evaluated their limitations, strengths, and key results. Then proceeded to identify common themes, trends, or patterns across the studies to develop a comprehensive understanding of the efficacy, safety, and other relevant outcomes of these therapies.

## 2. Pharmacological and Non-Pharmacological Treatments of HFpEF

HFpEF is currently diagnosed when a patient displays symptoms of HF and has a LVEF equal to or greater than 50%. HFpEF is primarily characterized by structural and functional abnormalities in the heart, leading to diastolic dysfunction and impaired ventricular filling. Due to the increasing life expectancy and the growing occurrence of cardiovascular risk factors, such as diabetes, obesity, or hypertension, the prevalence of HFpEF is projected to rise in the coming decades compared to HFrEF. It is estimated that HFpEF accounts for nearly 50% of all patients diagnosed with HF, which significantly impacts public health [5]. Unlike HFrEF or HFmrEF, the therapeutic strategies (angiotensin-converting enzyme inhibitors (ACEI), angiotensin receptor blockers (ARBs), mineralocorticoid receptor agonists (MRAs), β-blockers, calcium channel blockers (CCB)) have been debated in HFpEF. Therefore, optimal treatments for patients with HFpEF are urgently needed [6].

### 2.1 Conventional Medications in HFpEF

#### 2.1.1 ACEI, ARB, β-Blockers and CCB

Because HFpEF causes a significant amount of morbidity and mortality, there is an urgent need for effective treatments. ACEI, ARB, and β-blockers have not resulted in a reduction in morbidity or mortality in HFpEF unless prescribed for another comorbid disease. However, it has been shown that starting with both ACEI/ARB and β-blockers during hospitalization for HFpEF was associated with a lower risk of death from any cause for HF, as well as cardiovascular death compared with not starting with ACEI/ARB or β-blockers [7, 8].

Current evidence suggests that using CCB may improve outcomes in HFpEF patients. A post hoc data analysis of the Treatment of Preserved Cardiac Function Heart Failure with an Aldosterone Antagonist Trial (TOPCAT), which included 3440 HFpEF patients, with a mean follow-up period of 3.4 ± 1.7 years, showed that the all-cause mortality rate was lower in patients taking CCB than those not taking CCB (37.3 *vs*. 50.8 events per 1000 person-years, respectively). After adjusting for other factors, the hazard ratio for all-cause mortality was significantly lower in patients taking CCBs (hazard ratio (HR): 0.72, 95% confidence interval (95% CI): 0.59–0.88, *p* = 0.001). Similarly, cardiovascular and non-cardiovascular mortality risk was also lower (HR: 0.75, 95% CI: 0.59–0.96, *p* = 0.023 and HR: 0.68, 95% CI: 0.49–0.93, *p* = 0.018, respectively). Therefore, using CCB was associated with reduced mortality in patients with HFpEF, consistent with another 12-year prospective cohort follow-up study. However, the effectiveness of CCB, specifically for HFpEF patients, is still unknown. Therefore, current guidelines do not recommend the use of CCB for HFpEF patients [9, 10].

#### 2.1.2 Diuretics

Currently, diuretics are recommended for patients with HFpEF who have signs of fluid overload. It is essential to manage fluid retention by using diuretic treatment to improve the quality of life as a part of HFpEF therapy. According to the current guidelines, diuretics are necessary to alleviate symptoms in patients with HFpEF [11].

Spironolactone has been used in the treatment of HFpEF. It is associated with a slight reduction in hospitalizations related to HF. However, it does not reduce cardiovascular or overall mortality for patients with HFpEF [12]. In recent years, another detailed analysis of spironolactone therapy in the TOPCAT demonstrated spironolactone altered proteins and pathways such as caspase recruitment domain family member 18 (CARD18), polycystic kidney disease 2 (PKD2), pregnancy specific beta-1-glycoprotein 2 (PSG2), hepatocyte growth factor (HGF), phospholipid transfer protein (PLTP) and insulin-like growth factor 2 receptor (IGF2R), which were previously unknown but are affected by spironolactone in patients with HFpEF. These proteins and associated pathway analyses suggest that spironolactone influences caspase signaling, fibrosis, growth factors, and lipoprotein biology [13].

Furthermore, treatment with spironolactone is also effective for patients with HFpEF who have a high body mass index (BMI) and white blood cell (WBC) count. However, it can be detrimental for those with low BMI and alkaline phosphatase (ALP) levels. Therefore, a machine learning model can be used to develop improved, individual therapeutic plans in clinical trials [14].

Tolvaptan, a vasopressin V2 receptor antagonist, is a new diuretic approved in Japan for treating fluid retention in patients with HF. However, its effectiveness in patients with different subgroups of HF is still uncertain. A study aimed to investigate the safety and effectiveness of tolvaptan in different HF subgroups, involving 3349 patients treated with tolvaptan, and showed reductions in body weight and significantly improved congestive symptoms over the 14-day treatment period in the three HF subgroups. Therefore, tolvaptan is a safe and effective option for treating fluid retention in patients with the three HF subgroups [15]. To further investigate the long-term effectiveness of tolvaptan in treating HF, 591 patients were divided into HFpEF and HFrEF groups. The results demonstrated that the HFpEF group had a significantly lower all-cause mortality rate (38.6% compared to 24.7%) and a lower cardiovascular mortality rate during the 2.7-year follow-up. Multivariate analysis revealed that HFpEF was an independent factor influencing all-cause mortality (HR: 0.44, 95% CI: 0.23–0.86, *p* = 0.017). Thus, long-term use of tolvaptan may be more beneficial for HFpEF patients than for those with HFrEF [16]. The proposed mechanism is believed that tolvaptan can effectively manage fluid retention without activating the renin-angiotensin-aldosterone system (RAAS). Patients with HFpEF typically exhibit increased activity of the cardiac sympathetic nerves (CSN), contributing to a proportion of hospitalizations for acute decompensated heart failure (ADHF). Diuretic therapy is the primary treatment for relieving congestion in ADHF. However, conventional diuretic therapy may not necessarily improve prognosis due to the increased activity of the CSN. The effectiveness of tolvaptan in HFpEF may be attributed to its ability to relieve congestion without negatively affecting the neurohumoral systems in HFpEF patients [17].

#### 2.1.3 Angiotensin Receptor Neprilysin Inhibitors (ARNI)

In 2021, the Food and Drug Administration (FDA) approved sacubitril-valsartan (LCZ696) for treating HFpEF [18]. Studies indicated that sacubitril-valsartan treatment can reduce N-terminal pro-B-type natriuretic peptide (NT-proBNP) levels and improve cardiac function in patients with HFpEF and ADHF. No significant differences were observed in the rates of angioedema, hyperkalemia, worsening renal function, or symptomatic hypotension among these groups [19, 20]. These findings offer valuable insights into the clinical management of HFpEF and demonstrate that sacubitril-valsartan might be a practical approach for reducing HF events in patients with HFpEF [21].

With the exception of a few events, the PARAGON trial failed to show significant improvement with sacubitril-valsartan compared to valsartan in HFpEF patients. However, when the results from the PARADIGM-HF and PARAGON-HF trials were combined, it was demonstrated that sacubitril-valsartan significantly improved outcomes for patients with an ejection fraction of up to 55%. Additionally, another study have also examined the effectiveness and safety of sacubitril-valsartan in HFpEF patients and suggested that sacubitril-valsartan may reduce the risk of HF decompensation and all-cause mortality compared to valsartan [22]. However, there is still limited knowledge about its effects on HFpEF. Studies have shown that sacubitril-valsartan improved cardiac diastole function by reducing ventricular hypertrophy and fibrosis in mice. The potential underlying mechanism is that sacubitril-valsartan effectively suppresses the transmission of signals involved in calcium-mediated calcineurin-nuclear factor of activated T cell pathways [23].

### 2.2 New Disease-Modifying Treatment

#### 2.2.1 Treatment Targeting Epigenetic-Based Therapy

There is more emerging evidence that epigenetic regulation may have a role in the development of HF. The use of epigenetic-based therapy, also known as “epidrugs”, is gaining interest among the medical community. Targeting these epigenetic signals could be a promising treatment, especially for patients with HFpEF. Apabetalone is the first and only direct epidrug that has been tested on cardiovascular patients. The BETonMACE study has shown promising results regarding the use of apabetalone in reducing hospitalizations and cardiovascular deaths. Additionally, supplementing the diet with omega 3 polyunsaturated fatty acids (PUFAs) has been significantly linked to a reduced cardiovascular risk in both HFpEF and HFrEF. Further research is necessary to better understand and define specific epigenetic therapies that can selectively regulate transcriptional programs in certain types of cells [24, 25, 26, 27].

Additional investigations are needed to understand the mechanisms of epigenetic regulation. It has been found that gene alterations are associated with cardiovascular diseases, and researchers are constantly studying epigenetic mechanisms to develop new strategies to clinically manage HF patients.

#### 2.2.2 Treatment Targeting Ventricular Relaxation

Impaired relaxation of the ventricles affects the pressures of the left ventricle during exercise in patients with HFpEF. The function of sarcoplasmic/endoplasmic reticulum ${\mathrm{Ca}}^{2+}$-ATPase 2a (SERCA2a), which aids the relaxation of the myocardium by increasing calcium reuptake, is impaired in HFpEF. Istaroxime is a Na+/K+-ATPase inhibitor as well as a SERCA2a activator. Studies have shown that low doses of istaroxime did not impact the pressure of the heart or the relaxation parameters in patients with HFpEF during exercise. In contrast, higher doses of istaroxime are more effective in reducing exercise pulmonary capillary wedge pressure (PCWP) [28].

#### 2.2.3 Treatments Targeting Mitochondrial Abnormalities 

Growing evidence suggests mitochondrial abnormalities may be a significant factor in the development of HFpEF [29]. It has been proposed that hydrogen sulfide (H2S) plays a vital role in regulating mitochondrial function. A study investigating the protective effect of H2S on mitochondrial dysfunction in a mouse model of HFpEF showed that H2S improved left ventricular diastolic dysfunction by restoring mitochondrial abnormalities through the upregulation of peroxisome proliferator-activated receptor-gamma coactivator 1α (PGC-1α) and its downstream targets nuclear respiratory factor 1 (NRF-1) and mitochondrial transcription factor A (TFAM), which suggest that H2S supplementation has therapeutic potential in the treatment of multifactorial HFpEF [30].

Treatment strategies targeting the mitochondrial pathway to enhance the production of adenosine triphosphate (ATP) production may be advantageous for patients with HFpEF. Ubiquinol and D-ribose have been studied for their effects on increasing ATP synthesis within the mitochondria. Supplementation with these two substances may enhance the quality of life for patients with HFpEF. A 12-week treatment with 600 mg of ubiquinol or 15 g of D-ribose per day reduced HF symptoms, decreased levels of B-type natriuretic peptide (BNP) and lactate, and increased EF and ATP production [31].

Other studies aimed to identify and manipulate metabolic dysregulations in the myocardium of patients with HFpEF. We confirmed impaired gene expression in nicotinamide adenine dinucleotide (NAD)+ biosynthesis in the cardiac tissue of HFpEF patients. When HFpEF mice were supplemented with nicotinamide riboside or a direct activator of NAD+ biosynthesis, their mitochondrial function improved, and the HFpEF phenotype was alleviated. Therefore, these studies demonstrate that HFpEF is linked to dysfunction of the mitochondria in the myocardium and suggest that replenishing NAD+ levels is a promising therapeutic strategy for this condition. This research reveals for the first time, that mitochondrial dysfunction, specifically hyper-acetylation of mitochondrial proteins due to NAD+ deficiency, is likely a mechanism contributing to the pathogenesis of HFpEF. Therefore, targeting this common pathway could offer a new approach to treating HFpEF [32].

#### 2.2.4 Treatment Targeting Inflammation, Oxidative Stress and Nitrosative Stress 

Another effective method for treatment of HFpEF is to focus on long-term inflammation, which results in oxidative stress and microvascular dysfunction, which are significant factors in HFpEF [33, 34]. Myeloperoxidase (MPO) is an inflammatory enzyme primarily produced by neutrophils. Inhibiting MPO can decrease the production of free radicals, prevent dysfunction in small blood vessels, improve relaxation of heart muscle cells, reduce fibrosis, and potentially enhance heart function and clinical outcomes in patients with HFpEF. Therefore, it is a potential target for the therapeutic intervention of HFpEF against MPO [35]. AZD4831 is a novel, potent, and selective MPO inhibitor. It has a greater effectiveness in inhibiting extracellular MPO compared to intracellular MPO. This compound may also help maintain the protective function of intra-granular MPO in neutrophils. As a precursor drug, it can almost eliminate MPO activity. Studies have documented its pharmacokinetics, safety, tolerability, and target engagement in healthy volunteers and patients with HFpEF [36, 37].

Colchicine is used for treating inflammation and pain. Studies have shown that it can suppress nucleotide-binding oligomerization domain, leucine-rich repeat and pyrin domain-containing 3 (NLRP3) inflammation activation, which has been identified as an essential factor in HFpEF. Colchicine also improved heart function and reduced scar tissue in a heart failure model caused by a high-salt diet. Therefore, colchicine has the potential to be used as a treatment in patients with HFpEF [38, 39].

Recent studies have suggested that excessive production of nitric oxide (NO) through the enzyme inducible NO synthase (iNOS) contributes to the development of HFpEF. Moreover, dysfunction of Endothelial nitric oxide synthase (eNOS) and reduced availability of NO also contribute to the morbidity and mortality associated with HFpEF [40]. A study sought to investigate the dysregulation of NO signaling and the effects of a dual therapy using sodium nitrite and hydralazine in a mouse model of HFpEF. Treatment with sodium nitrite and hydralazine improved NO availability, reduced stress, improved endothelial function and mitochondrial respiration, limited fibrosis, and enhanced exercise capacity, ultimately mitigating the severity of HFpEF. The study showed a significant reduction in sodium nitrite and hydralazine, which suggested that combining NO-based therapeutics with a potent antioxidant and vasodilator agent could provide beneficial outcomes for treating HFpEF. However, further studies are needed to explore the role of NO in HFpEF and improve the understanding of NO-based therapies [41].

#### 2.2.5 Treatment Targeting Antidiabetic Drugs—Sodium-Glucose Co-Transporter 2 (SGLT2) Inhibitors, Glucagon-Like Peptide-1 (GLP-1) Receptor Agonists, Metformin and Dipeptidyl Peptidase-4 (DPP-4) Inhibitors

In addition to ARNI, treatment with SGLT2 inhibitors have demonstrated effectiveness in HFpEF. SGLT2 inhibitors could significantly reduce the risk of cardiovascular mortality and hospitalization in patients with HFpEF. Among SGLT2 inhibitors, empagliflozin, dapagliflozin, and canagliflozin are the most investigated. A double-blind trial investigating the effectiveness of empagliflozin in treating HFpEF, demonstrated that compared to the placebo group, patients in the empagliflozin group showed significantly lower primary adverse outcome events (13.8% *vs*. 17.1%). The hazard ratio was 0.79, with a 95% confidence interval of 0.69 to 0.90, indicating a significant difference (*p* < 0.001). This difference was primarily due to a lower risk of hospitalization for HF in the empagliflozin group. The total number of hospitalizations for HF was also lower in the empagliflozin group compared to the placebo group, with a hazard ratio of 0.73 and a 95% confidence interval of 0.61 to 0.88 (*p* < 0.001). Empagliflozin decreased the combined risk of cardiovascular mortality or hospitalization for HFpEF, in patients with and without diabetes [42, 43]. Among patients with HFpEF, dapagliflozin decreased the risk of total (first and recurring) HF events or cardiovascular mortality compared to a placebo. HF events can be prevented, and dapagliflozin reliably reduces the occurrence of these events, even in patients with different baseline medications [44]. In the DELIVER trial, which aimed to improve the lives of patients with HFpEF, dapagliflozin demonstrated a reduction in the risk of experiencing an overall rate of HF events, including both initial and subsequent HF hospitalizations, worsening HF events for the first time as well as cardiovascular death. This reduction was also observed in patients with HFpEF [45].

Studies have shown that canagliflozin therapy reduced blood pressure, body weight, and cardiac remodeling, as well as improved left ventricular diastolic dysfunction in a rodent model of HFpEF. Additionally, treatment with canagliflozin also reduced myocardial hypertrophy and fibrosis. These positive changes are probably related to activating the angiotensin-converting enzyme 2 (ACE2) and increasing levels of ACE2/Ang(1-7)/G-protein coupled receptor (MAS receptor) axis, as well as mitigating ferroptosis via blocking iron overloading and lipid peroxidation. Therefore, canagliflozin is a promising agent for preventing and treating HFpEF [46], however more clinical data are needed.

In addition to SGLT2 inhibitors, GLP-1 receptor agonists are used to treat diabetes and have been shown to positively affect heart and kidney diseases. Data is available on their potential benefits of SGLT2 inhibitors in HFpEF. However, there is not enough evidence available for GLP-1 receptor agonists. To investigate the impact of GLP-1 receptor agonists on HF, in patient with and without diabetes, a systematic review and meta-analysis of randomized controlled trials focused on HF hospitalization, cardiac function, and structural changes. GLP-1 receptor agonists did not reduce HF readmissions in patients with a history of HF and elevated NT-proBNP levels. Therefore, the prognosis of HF did not improve despite a significant improvement in left ventricular diastolic function. Further research is still needed to explore the effects of GLP-1 receptor agonists on cardiac function and the prognosis of patients with HFpEF [47].

Observational studies suggest that metformin may provide a mortality benefit in individuals with HF. However, whether metformin has any benefits for individuals with HFpEF remains unexplored. To address this, we conducted a systematic review and meta-analysis to determine if the variation in EF affects mortality outcomes in HF patients treated with metformin. Metformin was found to reduce mortality in patients with both HFpEF and HFrEF. Significant protective effects were observed in patients with an EF greater than 50% (*p* = 0.003). Metformin treatment combined with insulin, ACEI, and β-blocker therapy also reduced mortality (insulin *p* = 0.002; ACEI *p* < 0.001; β-blocker *p* = 0.017). Female gender was associated with worse outcomes (*p* < 0.001). Therefore, treatment with metformin is related to a reduction in mortality for patients with HFpEF [48].

There is a debate regarding whether DPP-4 inhibitors should be the preferred glucose-lowering agents for individuals with HFpEF. A study aimed to assess the effects of DPP-4 inhibitors treatment on mortality and cardiovascular outcomes in HF patients. Subgroup analyses revealed that DPP-4 inhibitors significantly decreased all-cause mortality in trials with more than 40% of female patients (HR: 0.30, 95% CI: 0.16–0.58, *p* = 0.0003) and in trials with more than 20% of patients with HFpEF (HR: 0.34, 95% CI: 0.19–0.60, *p* = 0.0003). DPP-4 inhibitors may reduce all-cause mortality in specific subgroups of HF patients, especially in women and those with HFpEF, while maintaining a favorable coronary safety profile. Additionally, the use of DPP-4 inhibitors was found to be associated with improved long-term outcomes in HFpEF patients with diabetes mellitus [49, 50].

### 2.3 Non-Pharmacologic Treatment

#### 2.3.1 Aortic Thoracic Stimulation with the Harmony™ System 

Currently, available medical treatments for HFpEF are insufficient, which has led to the development of device-based solutions that target the underlying abnormalities causing this condition. HFpEF is characterized by an autonomic imbalance, specifically a decrease in vagal parasympathetic activity and an increase in sympathetic signaling. This imbalance contributes to the deterioration of cardiac performance and an overall increase of morbidity and mortality. It has been suggested that stimulating the thoracic aortic vagal afferents can restore autonomic balance. The Harmony™ System (Enopace, Israel) is a neuromodulation platform implanted in patients. It comprises an implantable unit, delivery catheter, patient wearable unit, and programming unit. In this study, chronic stimulation of aortic vagal afferents using this system has been shown to improve left atrial remodeling and function, as well as left ventricular performance, highlighting the potential of neuromodulation therapy as an effective treatment for HFpEF [51].

#### 2.3.2 Percutaneous Interatrial Shunting Devices

An increase in the left atrial pressure is the main cause of pulmonary congestion, which leads to difficulty breathing and limited exercise capacity. There is limited evidence on the benefits of medical treatment for patients with HFpEF. Therefore, alternative strategies have been developed, including devices that can lower the left atrial pressure by creating communications between the left and right atria, causing a shunt. Techniques to create an intentional shunt from the left to the right atrium were developed, which lowers left atrium pressure, reduces pressure on the pulmonary circulation, and decreases pulmonary congestion. Interatrial septal devices offer a new approach to treat HFpEF by targeting the result of increased left atrial pressure instead of focusing on the complex pathways leading to its development. Early pilot studies and randomized trials have shown that these devices are safe and effective. The pivotal Inter Atrial Shunt Device (IASD) trial has now completed enrollment and is in the follow-up phase [52]. The ideal selection of patients should have a confirmed diagnosis of HFpEF and symptoms, such as shortness of breath during exertion, exercise intolerance, fatigue, fluid retention; increased pressures in the left atrium, which can be assessed using echocardiography, cardiac catheterization, and other diagnostic tools.

#### 2.3.3 Percutaneous Radiofrequency Ablation–Based Interatrial Shunting

Another study evaluated the safety and effectiveness of using percutaneous radiofrequency ablation to create an interatrial shunt for HFpEF using a new atrial septostomy device. A preclinical study was conducted using 11 normal domestic pigs, followed by a study involving 10 patients with HFpEF. The percutaneous radiofrequency ablation-based interatrial shunting therapy was successfully performed in both animals and patients. No significant safety events, including death and thromboembolism, were observed. The clinical status significantly improved, with NT-proBNP reduced by 2149 pg/mL (95% CI: 204–3301, *p* = 0.028), six-minute walk test (6MWT) increased by 88 m (95% CI: 50–249, *p* = 0.008), and New York Heart Association (NYHA) classification improved in eight patients after 6 months. These findings suggest that percutaneous radiofrequency ablation-based interatrial shunting is a safe and potentially effective therapy for HFpEF, providing a non-pharmacological and non-implanted option for managing HFpEF [53].

#### 2.3.4 Cardiac Contractility Modulation

Cardiac Contractility Modulation (CCM) is an incremental treatment that includes enhanced peak oxygen uptake, NYHA classification, health status, and the difference in 6MWT. A study sought to assess the effectiveness and safety of CCM. Exercise training programs enhance the cardiorespiratory performance of patients with HFpEF, showing an increase in peak oxygen uptake (${\mathrm{VO}}_{\text{2peak}}$), 6MWT and the ventilatory threshold. The results indicate that CCM therapy could significantly enhance the health status of HFpEF while maintaining the same safety profile observed in patients receiving CCM therapy for systolic dysfunction. Thus, it is crucial to encourage the prescription of exercise training programs for HFpEF patients [54].

#### 2.3.5 Cardiac Rehabilitation

The objectives of cardiac rehabilitation in patients with HFpEF generally consist of enhancing exercise capacity, optimizing medication therapy, and promoting a healthy lifestyle. Here are some essential components of cardiac rehabilitation for individuals with HFpEF:

Exercise Training: HFpEF patients should initiate an exercise training program with shorter intervals and progressively extend the duration of each interval as their exercise capacity improves. In clinically, the exercise training (cycling, walking) for stable HFpEF patients is recommended for 45 to 60 min, 3 to 5 d/wk at a moderate to high-intensity to improve ${\mathrm{VO}}_{\text{2peak}}$ [55].

Education and counseling: education plays a significant role in helping HFpEF patients comprehend their condition, which include providing information regarding heart-healthy diets, medication adherence, symptom recognition, and stress management techniques.

Medication optimization, risk factor modification and psychosocial support: cardiac rehabilitation programs may collaborate with healthcare providers to optimize medication therapy for HFpEF patients. Risk factor modification may include quitting smoking, maintaining a healthy weight, controlling blood pressure, glucose and lipid levels. Emotional well-being and social support are vital aspects of cardiac rehabilitation for HFpEF patients as well.

## 3. Pharmacological and Non-Pharmacological Treatments of HFrEF

HFrEF is a complex and progressive medical condition characterized by shortness of breath and functional impairment. Because of its high mortality and morbidity, it is one of the most significant challenges in public health. The main pharmacological treatment strategies include using a combination of medications (ARB/ARNI, β-blockers, mineralocorticoid receptor antagonists, and SGLT2 inhibitors) to reduce hospitalizations, all-cause mortality, and cardiovascular mortality. Additional novel medications such as soluble guanylate cyclase stimulators, direct myosin activators, selective sinus-node inhibitors, iron supplements and Chinese medicine, may also be helpful for patients with HFrEF.

### 3.1 Evidence for Fundamental Pharmacological Treatments

#### 3.1.1 ACEI, ARB

Harmful upregulation of the RAAS is involved in HF progression, resulting in fluid retention, peripheral arterial vasoconstriction, cardiomyocyte hypertrophy, interstitial fibrosis, and heart remodeling. A study has shown that ACEI acting as a RAAS antagonist, reduced hospitalization and mortality in HFrEF [56]. The SOLVD study showed that in HF patients with LVEF ≤35%, enalapril reduced total death risk and cardiovascular death risk by 16% (95% CI: 0.05–0.26, *p* = 0.0036) and 18% (95% CI: 0.06–0.28, *p* = 0.002), respectively. Enalapril also reduced the rate of HF hospitalization by 26% (95% CI: 0.18–0.34, *p* < 0.0001). However, enalapril caused an increase in dizziness or fainting and cough, a significant decrease in blood pressure, and small but statistically significant increases in serum potassium and creatinine levels [57, 58].

Similar to ACEI, ARB can also combat RAAS. Long-term application of ARB in HFrEF patients improves hemodynamics and reduces cardiovascular disease (CVD) and rates of HF hospitalization [59, 60, 61]. ARB is better tolerated in patients who cannot tolerate ACEI. The CHARM-Added trial investigated whether combining the two medication improves clinical outcomes in HFrEF. All subjects with NYHA class II–IV and LVEF ≤40% treated with ACEI were randomly assigned to the candesartan or the placebo group. Candesartan significantly reduced the primary outcome, the risk of the composite outcome of CVD or HF hospitalization rate to 85% (95% CI, 0.75–0.96; *p* = 0.011). The Val-HeFT study showed that valsartan reduced the combined endpoint of mortality and morbidity by 13.2% (relative risk (RR): 0.87, 97.5% CI: 0.77–0.97, *p* = 0.009) and significantly improved NYHA class, EF, signs and symptoms of HF, and quality of life [62].

#### 3.1.2 ARNI

LCZ696, a novel dual-acting inhibitor of the angiotensin II receptor and neprilysin, is used to block natriuretic peptides and RAAS, thereby potentially favoring the modulation of neurohormone imbalances characteristic of HF [63]. Studies have shown that sacubitril/valsartan significantly reduced the composite outcome of CVD or HF hospitalization rate and NT-proBNP levels in patients with HFrEF [64, 65].

The PARADIGM-HF trial compared sacubitril-valsartan and enalapril’s long-term efficacy and safety in patients with HFrEF [63]. 8442 patients with NYHA functional class II–IV and LVEF ≤35% were included with a composite endpoint of CVD or HF hospitalization rate as the primary outcome. Compared with enalapril, sacubitril/valsartan significantly reduced the primary outcome (HR: 0.80, 95% CI: 0.73–0.87, *p* < 0.001) and reduced symptoms and physical limitations in HF (*p* = 0.001) [64]. Sacubitril-valsartan was comparable to valsartan in reducing NT-proBNP. In the LIFE trial, the effects of sacubitril-valsartan and valsartan on NT-proBNP were compared in patients with advanced heart failure and NYHA class IV and LVEF ≤35%. There was no statistical significance between the two drugs (*p* = 0.45) [65]. Although sacubitril-valsartan caused higher rates of hypotension and angioedema; renal impairment, hyperkalemia, and rate of coughing were lower than enalapril. Furthermore, it did not increase dementia-related side-effects [66].

#### 3.1.3 β-blockers

β-blockers slow heart rate by inhibiting sympathetic excitation. Thus, cardiomyocyte metabolism may be improved by conserving energy, improving calcium recycling, increasing diastolic blood flow, and preventing ischemia [67]. Furthermore, β-blockers were able to reduce mortality in patients with HFrEF. A study showed that three β-blockers, bisoprolol, carvedilol, and metoprolol, had similar effects on HF mortality. There was no significant association between β-blocker selection and all-cause mortality in any of the matched samples (*p* > 0.05 for bisoprolol *vs*. carvedilol, bisoprolol *vs*. metoprolol succinate, and carvedilol *vs*. metoprolol succinate) [68]. Another study compared the tolerance of bisoprolol *vs*. carvedilol in patients with HFrEF, defined as achieving and maintaining the maximum maintenance dose during 48 weeks of treatment. The primary endpoint was achieved in 41.4% of the bisoprolol group and 42.5% of the carvedilol group, a nonsignificant difference (*p* = 0.0899). The Carvedilol group had a more significant decrease in BNP and a smaller decrease in heart rate [69].

#### 3.1.4 Mineralocorticoid Receptor Antagonists (MRAs)

MRAs contributed to blocking RAAS, and studies have confirmed that MRAs can reduce mortality and hospitalization rates in patients with HFrEF. Notably, MRAs have the effect of elevating blood potassium. The EMPHASI-HF trial investigated MRAs, eplerenone, and its effect on clinical outcomes in patients with NYHA class II and LVEF ≤35%. The composite outcome of CVD or HF hospitalization rates occurred in 18.3% of the eplerenone group *vs*. 25.9% of the placebo group (HR: 0.63, 95% CI: 0.54–0.74, *p* < 0.001). The eplerenone group also had lower rates of all-cause mortality, CVD, HF hospitalization rates, and hospitalization for any cause than the placebo group [70]. Another clinical registry study, the COMPARE-HF trial, also confirmed that MRAs can reduce the risk of re-hospitalization in patients with HFrEF. Among patients with NYHA class II–IV and LVEF ≤35%, those treated with MRAs had a lower rate of HF readmission at 3 years than those treated with placebo (HR: 0.87, 95% CI: 0.77–0.98, *p* = 0.02) [71].

### 3.2 New Disease-Modifying Treatments

#### 3.2.1 Treatment Targeting Soluble Guanylate Cyclase (sGC) Stimulator

Vericiguat, a novel oral sGC stimulator, enhances the cyclic guanosine monophosphate (cGMP) pathway by directly stimulating sGC through a binding site independent of NO. It sensitizes sGC to endogenous NO by stabilizing NO binding. A study has shown that vericiguat can directly dilate blood vessels, prevent fibrosis, improve myocardial compliance and diastolic function, improve endothelial function, and improve myocardial remodeling [72]. Vericiguat effectively reduced the composite outcome of CVD or HF hospitaliztion in HFrEF patients with a good safety profile [73, 74]. The VICTORIA trial was a randomized, placebo-controlled, parallel-group, multicenter, double-blind, event-driven phase 3 trial that evaluated the efficacy and safety of vericiguat in HFrEF [72]. The study enrolled 5050 patients with HF (NYHA functional class II-IV) and an LVEF <45% who received either vericiguat or placebo, in addition to guideline-based medical therapy. Rates of CVD or HF hospitalization were lower with vericiguat than with placebo (HR: 0.90, 95% CI: 0.83–0.98, *p* = 0.02) [73]. The rates of safety events were low and similar in the two groups (adjusted HR: 1.18, 95% CI: 0.99–1.39, *p* = 0.059). In patients prone to hypotension, the use of vericiguat initially decreased blood pressure, but pressures subsequently returned to baseline levels [74]. Unfortunately, vericiguat did not significantly improve health outcomes compared to placebo. Health status outcomes were represented by the Kansas City Cardiomyopathy Questionnaire (KCCQ). The Clinical Summary Score (CSS), Total Symptom Score (TSS), and Overall Summary Score (OSS) were calculated separately. There was no significant difference in the improvement of CSS, TSS, and OSS between the two groups at 4, 16, and 32 weeks (*p* > 0.05) [75].

As to HFpEF, there are two main clinical trials have been published to test vericiguat in patients with HFpEF: the phase II study SOCRATES–PRESERVED and the phase III study VITALITY. The phase II study SOCRATES-PRESERVED, aimed to analyze the safety, tolerability, and pharmacological properties vericiguat over a period of 12 weeks. The results demonstrated that there was no significant difference in the vericiguat treatment group compared to the placebo group. On the other hand, the VITALITY study focused on evaluating the effectiveness and safety of vericiguat on patients’ quality of life and exercise tolerance. After 24 weeks of treatment, there was no significant difference between vericiguat treatment group and the placebo group regarding to KCCQ scores or the results of the 6MWT [76, 77].

#### 3.2.2 SGLT2 Inhibitors

In patients with type 2 diabetes, SGLT2 inhibitors reduced the rates of HF hospitalization and the risk of serious adverse renal events, a benefit not associated with other hypoglycemic agents [78, 79]. These benefits were most pronounced in patients with HFrEF but could not be explained by the effect of lowering blood glucose. Consistent with these observations, SGLT2 inhibitors reduce the incidence of the composite outcome of CVD or HF hospitalization rates in patients with HFrEF, regardless of diabetes, and have a low risk of severe renal outcomes [80, 81]. In addition, relevant studies have shown that SGLT2 inhibitors can improve cardiac remodeling, hemodynamics, and anemia [82, 83].

In the EMPEROR-Reduced randomized trial, empagliflozin significantly reduced the incidence of a combined outcome of CVD and HF hospitalization rates compared to placebo, regardless of whether the patients had diabetes (HR: 0.75, 95% CI: 0.65–0.86, *p* < 0.001). In secondary outcomes, the total number of HF hospitalization rates was lower in the empagliflozin group than in the placebo group. The annual rate of decline in the estimated glomerular filtration rate was slower than with placebo. It was associated with a lower risk of severe renal outcomes [80]. The Empire HF trial investigated the effects of empagliflozin on cardiac remodeling and hemodynamics in HFrEF. In cardiac remodeling, empagliflozin significantly reduced the left ventricular end-systolic volume (LVESV) index (–4.3 mL/$m^{2}$; *p* = 0.04), left ventricular end-diastolic volume (LVEDV) index (–5.5 mL/$m^{2}$; *p* = 0.03), and left atrial volume index (–2.5 mL/$m^{2}$; *p* = 0.04) [82]. Empagliflozin significantly reduced PCWP (–2.40 mmHg; *p* = 0.003) [83]. In addition, empagliflozin significantly reduced stressed blood volume by 9% (*p* = 0.001). However, there was no change in NT-proBNP levels (*p* = 0.7), daily activity level (*p* = 0.4), and health status (*p* = 0.6) [84].

Dapagliflozin is another commonly used SGLT2 inhibitor. The DAPA-HF trial is a phase 3 placebo-controlled trial, a composite of worsening HF (hospitalization or an urgent visit resulting in intravenous therapy for HF) or CVD as the primary outcome. The results showed that dapagliflozin reduced the incidence of the primary outcome in patients with HFrEF (HR: 0.74, 95% CI: 0.65–0.85, *p* < 0.001) regardless of the presence of diabetes. It also reduced worsening HF events (HR: 0.70, 95% CI: 0.59–0.83) and incidence of CVD (HR: 0.82, 95% CI: 0.69–0.98). Similar results were obtained in subgroup analyses of sex, age, frailty status, and glycated hemoglobin [85, 86, 87]. Moreover, dapagliflozin significantly improved the health status of the patients, manifested as improving TSS, CSS, and OSS scores *vs*. placebo (*p* < 0.0001) [88].

#### 3.2.3 Treatment Targeting Direct Myosin Activators

Omecamtiv mecarbil (OM), a novel direct myosin activator, has improved cardiac function and reduced the risk of CVD or first HF events in patients with HFrEF [89, 90].

The COSMIC-HF trial assessed the effects of OM on cardiac function and structure. For the OM group *vs*. the placebo group, least square mean differences were as follows: systolic ejection time, 25 ms (95% CI: 18–32, *p* < 0.0001); stroke volume, 3.6 mL (95% CI: 0.5–6.7, *p* = 0.0217); left ventricular end-systolic diameter (LVESD), –1.8 mm (95% CI: –2.9 to –0.6, *p* = 0.0027); left ventricular end-diastolic diameter (LVEDD), –1.3 mm, (95% CI: –2.3 to 0.3, *p* = 0.0128), heart rate, –3.0 beats/min (95% CI: –5.1 to –0.8, *p* = 0.007); and NT-proBNP, –970 pg/mL (95% CI: –1672 to –268, *p* = 0.0069) [89]. The GALACTIC-HF trial analyzed the efficacy and safety of OM in the treatment of HF. A total of 8232 HFrEF patients with NYHA class II–IV and LVEF ≤35% were enrolled in this study, in which NYHA class III–IV and LVEF ≤30% were defined as severe HF. OM was associated with a significant benefit *vs*. placebo in patients with severe HF (HR: 0.80, 95% CI: 0.71–0.90, *p* < 0.001), but not in those with non-severe HF (HR: 0.99, 95% CI: 0.91–1.08, *p* = 0.84). OM was well tolerated in patients with severe HF, with no significant changes in blood pressure, renal function, or potassium levels [90]. However, a relevant study did not support the effect of OM on improving the exercise capacity of HFrEF patients [91].

#### 3.2.4 Treatment Targeting Iron Supplements

Iron is crucial in oxygen uptake, transport, storage, and oxidative metabolism in skeletal muscle. It also participates in erythropoiesis. Patients with HF may be susceptible to iron deficiency due to depleted iron stores or defective iron absorption and reduced availability of circulating iron in the reticuloendothelial system [92]. Intravenous ferric carboxymaltose (FCM) has been shown to improve symptoms, functional capacity, health status, and quality of life in iron-deficient patients with HFrEF [93].

The CONFIRM-HF trail had 6MWT as the primary endpoint, and FCM significantly prolonged the 6MWT distance (difference FCM *vs*. placebo: 33 ± 11 m; *p* = 0.002). In addition, patients treated with FCM significantly improved in NYHA class and fatigue scores. Mortality and adverse event rates were similar in the FCM and placebo groups [92]. A pooled analysis of the effects of FCM on the health status of patients with HFrEF showed that the average KCCQ and OSS improvement score was higher in the FCM group than in the placebo group, and a higher proportion of patients showed improvement. This improvement was sustained for more than half a year [93]. Unfortunately, inconsistent with FCM, oral iron supplementation failed to improve exercise capacity and quality of life [94].

#### 3.2.5 Treatment Targeting Chinese Medicine

Traditional Chinese medicine is an alternative treatment for HF. The Qiliqiangxin capsule (QLQX) is a traditional Chinese medicine preparation widely used in treating HF in China. It has been included in the Chinese guidelines for diagnosing and treating HF as a clinical recommendation for HFrEF. A multicenter, randomized, placebo-controlled trial with reduced NT-proBNP levels as the primary endpoint enrolled 512 patients. The results showed that QLQX combined with standard therapy significantly reduced NT-proBNP levels compared with the control group (*p* = 0.002). In secondary outcomes, QLQX showed superior performance in improving NYHA class, LVEF, 6MWT distance, and quality of life [95]. Another randomized controlled trial has demonstrated that QLQX is safer and more effective than placebo and the standard of care in treating HF, the potential protective mechanism is probably related to reducing myocardial apoptosis and improving cardiac function [96].

### 3.3 Nonpharmacologic Therapy for HFrEF

#### 3.3.1 Cardiac Resynchronization Therapy (CRT) 

With CRT, symptoms and quality of life of patients with HF and cardiac dyssynchrony can be improved and the risk of complications and death can be reduced [97]. It has now been added to the Chinese guidelines for diagnosing and treating HF as an essential complementary treatment modality. Catheter ablation (CA) combined with CRT is more effective than medication in lowering the risk of death in patients with permanent atrial fibrillation and narrow electrocardiographic QRS wave caused by HF, regardless of their initial cardiac EF. A study discovered that resynchronization of the left bundle branch was particularly beneficial in enhancing LVEF among patients with non-ischemic cardiomyopathy and HF with left bundle branch block [98]. In patients with mild HF symptoms, LVEF below 30%, QRS duration equal to or greater than 130 ms, and cardiomyopathy, CRT successfully decreased LVEDV and improved long-term survival after one year [99].

#### 3.3.2 Baroreflex Activation Therapy (BAT)

BAT is an innovative treatment for patients with HF. It contains a device implantation that electrically stimulates the baroreceptors, which are specialized sensors located in the carotid arteries. The objective of this therapy is to enhance heart function and alleviate symptoms by regulating the body’s autonomic nervous system. BAT reduces sympathetic tone and enhances vagal tone through a central nervous system (CNS)-mediated mechanism, which transmits stimulated electrical energy to the carotid sinus via wires, which can help improve cardiac function in patients with HFrEF and enhance their clinical prognosis. Studies showed that BAT safely improved HFrEF patients’ quality of life, exercise capacity, NT-proBNP concentration, heart rate, and blood pressure levels [100, 101]. A meta-analysis also showed that BAT enhances exercise performance, NYHA ratings, and quality of life in HF patients treated with guideline-directed medication therapy (GDMT) [102].

#### 3.3.3 Cardiac Rehabilitation 

Cardiac rehabilitation refers to a series of comprehensive measures, including exercise therapy, lifestyle modification, and psychological intervention, to give physiological and psychological support to patients with acute and chronic CVD. A study focused on left ventricular systolic function in patients with stage B HF after one or more weekly cardiac rehabilitation treatments for 6 months after hospital discharge, to evaluate to effects of cardiac rehabilitation on the composite end-point of all-cause mortality and HF rehospitalization rates over a two-year follow-up period. The results demonstrated that cardiac rehabilitation was associated with a composite risk reduction (HR: 0.66, 95% CI: 0.48–0.91, *p* = 0.011), including all-cause mortality (HR: 0.53, 95% CI: 0.30–0.95, *p* = 0.032), and HF re-hospitalization (HR: 0.66, 95% CI: 0.47–0.92; *p* = 0.012) [103].

Cardiac rehabilitation treatments consist of medication, exercise, nutrition, psychology, and smoking cessation. Participating in exercise-based cardiac rehabilitation improves the patient’s exercise capacity and quality of life [104].

## 4. Pharmacological Treatments of HFmrEF

HF with mid-range EF (40–49%) has been renamed as “HF with mildly reduced EF” (HFmrEF) due to its distinct similarities with HFrEF. According to the 2021 European guidelines, β-blockers, MRAs, RAAS inhibitors, and ARNI, SGLT2 inhibitors might be used for patients with HFmrEF, but with a IIB class of recommendation. Many large-scale clinical trials on ARNIs, MRAs, ACE inhibitors, ARBs, and β-blockers demonstrated neutral results in the primary composite outcome of death or HF hospitalization for patients with HFmrEF. Nevertheless, these drug classes have been recommended to treat HFmrEF by US guidelines because of their marginal benefits regarding HF hospitalization [4, 105].

## 5. Limitations and Prospects

Despite making progress in diagnosing, treating, monitoring, and improving outcomes for HF, there are still significant needs that remain unaddressed. These include the need for new biomarkers. Natriuretic peptides and cardiac troponins may not directly show the mechanisms behind the increased risk of arrhythmias, such as issues with calcium handling. Therefore, it is necessary to find new biomarkers that can predict the remaining risk that conventional biomarkers do not capture. Additionally, more studies directly comparing different treatment options for HF are needed.

In the future, with advancements in genetics, molecular biology, and imaging techniques, there is an increasing emphasis on personalized, more accurate and efficient therapies. Additionally, the management of HF typically requires a team of healthcare professionals such as cardiologists, nurses, pharmacists, cardiac rehabilitation physicians and other healthcare professionals, which can improve communication, coordination, and patient outcomes.

Ongoing clinical trials, technological advancements, and new discoveries will influence the future development of diagnosing, treating, and managing HF patients. By addressing these limitations and exploring further research, we can improve our understanding and management of HF.

## 6. Conclusions

Heart failure remains a predominant cause of public health issues. Despite advancements in pathological mechanisms and therapies, mortality and morbidity remain high, especially among patients above 65 years of age. A poor quality of life and severe economic burden is also increasing annually. Therefore, efficient strategies are still urgently needed. We summarize three HF subgroups’ pharmacological and non-pharmacological advancements targeting conventional and novel therapies. Diuretics, SGLT2 inhibitors, and ARNI are the commonly used medications in patients with three subgroups. It will be important to evaluate whether novel therapies offer any additional benefits compared to current treatment options, such as better outcomes, fewer side effects, or improved patient adherence. The economic implications of using the novel therapies in clinical practice will need to be completely analyzed. Additionally, long-term safety and any specific risks associated with therapies will need to be considered. The deeper we investigate the etiology and pathophysiology of HF, the more we need to explore novel, safety and effective strategies of HF.

## Abbreviations

HF, heart failure; LVEF, left ventricular ejection fraction; HFpEF, heart failure with preserved ejection fraction; HFrEF, heart failure with reduced ejection fraction; HFmrEF, heart failure with mildly reduced ejection fraction; ACEI, angiotensin-converting enzyme inhibitors; ARB, angiotensin-II receptor blockers; PUFAs, polyunsaturated fatty acids; BMI, body mass index; WBC, white blood cell; ALP, alkaline phosphatase; CSN, cardiac sympathetic nerves; ADHF, acute decompensated heart failure; MRAs, mineralocorticoid receptor agonists; CCB, calcium channel blockers; TOPCAT, treatment of preserved cardiac function heart failure with an aldosterone antagonist trial; ARNI, angiotensin receptor neprilysin inhibitors; FDA, food and drug administration; eNOS, endothelial nitric oxide synthase; SERCA2a, sarcoplasmic/endoplasmic reticulum ${\mathrm{Ca}}^{2+}$-ATPase 2a; H2S, hydrogen sulfide; PGC-1α, peroxisome proliferator-activated receptor-gamma coactivator 1α; NRF-1, targets nuclear respiratory factor 1; TFAM, mitochondrial transcription factor A; ATP, adenosine triphosphate; BNP, B-type natriuretic peptide; MPO, myeloperoxidase; SGLT2, sodium-glucose co-transporter 2; GLP-1, glucagon-like peptide-1; DPP-4, dipeptidyl peptidase-4; ACE2, angiotensin-converting enzyme 2; LVEDV, left ventricular end-diastolic volume; LVESV, left ventricular end-systolic volume; LVESD, left ventricular end-systolic diameter; LVEDD, left ventricular end-diastolic diameter; IASD, inter atrial shunt device; NT-proBNP, N-terminal pro-B-type natriuretic peptide; CCM, cardiac contractility modulation; 6MWT, 6-min walk test; NYHA, New York Heart Association; ${\mathrm{VO}}_{\text{2peak}}$, peak oxygen uptake; RAAS, renin-angiotensin-aldosterone system; CVD, cardiovascular disease; sGC, soluble guanylate cyclase; cGMP, cyclic guanosine monophosphate; KCCQ, Kansas City Cardiomyopathy Questionnaire; CSS, clinical summary score; TSS, total symptom score; OSS, overall summary score; PCWP, pulmonary capillary wedge pressure; OM, omecamtiv mecarbil; FCM, ferric carboxymaltose; CRT, cardiac resynchronization therapy; CA, catheter ablation; BAT, baroreflex activation therapy; GDMT, guideline-directed medication therapy.
